# Spatio-temporal co-occurrence of hotspots of tuberculosis, poverty and air pollution in Lima, Peru

**DOI:** 10.1186/s40249-020-00647-w

**Published:** 2020-03-24

**Authors:** Gabriel Carrasco-Escobar, Alvaro Schwalb, Kelly Tello-Lizarraga, Percy Vega-Guerovich, Cesar Ugarte-Gil

**Affiliations:** 1 Health Innovation Lab, Instituto de Medicina Tropical Alexander von Humboldt, Universidad Peruana Cayetano Heredia, Lima, Peru; 2 Division of Infectious Diseases, Department of Medicine, University of California San Diego, La Jolla, CA USA; 3 Instituto de Medicina Tropical Alexander von Humboldt, Universidad Peruana Cayetano Heredia, Lima, Peru; 4 School of Public Health and Administration, Universidad Peruana Cayetano Heredia, Lima, Peru; 5 School of Economics and Finance, Universidad del Pacifico, Lima, Peru; 6 School of Medicine, Universidad Peruana Cayetano Heredia, Lima, Peru; 7 TB Centre, London School of Hygiene and Tropical Medicine, London, UK

**Keywords:** Tuberculosis, Hotspots, air pollution

## Abstract

Growing evidence suggests pollution and other environmental factors have a role in the development of tuberculosis (TB), however, such studies have never been conducted in Peru. Considering the association between air pollution and specific geographic areas, our objective was to determine the spatial distribution and clustering of TB incident cases in Lima and their co-occurrence with clusters of fine particulate matter (PM_2.5_) and poverty. We found co-occurrences of clusters of elevated concentrations of air pollutants such as PM_2.5_, high poverty indexes, and high TB incidence in Lima. These findings suggest an interplay of socio-economic and environmental in driving TB incidence.

## Background

In 2017, 10 million new cases of tuberculosis (TB) occurred worldwide [1] which constitute a major health burden that strains middle- and low-income countries. Many socio-economic factors within these countries are frequently associated with higher TB incidence such as poverty, unemployment, low income, overcrowding, and population density [2]. It is well known that TB is prone to spatial aggregation often in poor areas of cities and can even be associated with a higher risk of infection, as observed in Southern Ethiopia where the risk is 4.16 times higher inside a cluster [3]. The use of geographical surveillance in public health allows for the detection of areas with a high prevalence or incidence of a particular disease in order to identify socio-economic factors associated with the phenomenon [4]. These methods have been applied to TB transmission [5]. Spatial information contributes to appropriate decision-making with a more efficient budget and human resources allocation and has been used previously in infectious diseases to detect hotspots and epidemics [6].

Environmental factors such as pollution and suspended particles are considered to play important roles in the development of TB [7]. This is explained by secretion clearance impairment by epithelial cells of the respiratory tract which is the primary defense mechanism against *Mycobacterium tuberculosis* [8].

Studies on spatio-temporal distribution of TB cases have principally addressed their association with demographic and geographical predictors for multidrug-resistant TB in Peru [9–11], nonetheless, their possible association with environmental factors such as air pollution have yet to be of interest. This descriptive study sought to determine the spatial distribution and clustering of TB cases in Lima, Peru and determine co-occurrence with clusters of PM_2.5_ and economic index.

## Methods

### Study design

Ecological analysis using Peruvian Ministry of Health (MoH) data of TB incidence cases from 2015 to 2017 and high-resolution fine particulate matter with aerodynamic diameter of 2.5 μm or smaller (PM_2.5_) from the National Aeronautics and Space Administration (NASA) [12].

### Study area and population

**Fig. 1:**
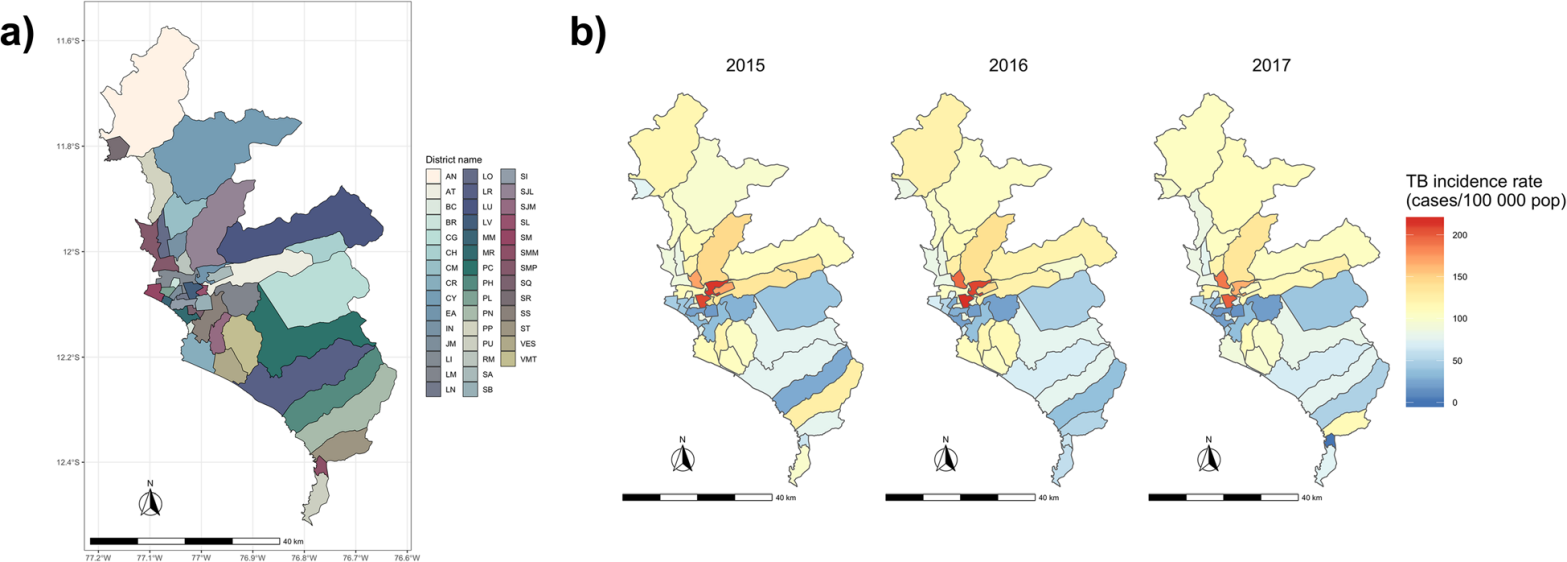
Study area in Lima Province, Peru. **a** Districts in Lima, each color represents a district. **b** Tuberculosis incidence rate (cases/100 000 pop) in Lima, 2015–2017

This study was conducted in Lima, Peru. Lima has a population of almost 9 million in an area of 2672 km^2^. It is divided in 43 districts (Fig. 1a), some of which are the most densely populated districts in Peru. Overall, Lima is the most developed province in the country with the largest internal migration rate.

### Data sources

#### Tuberculosis cases

All new TB cases are reported in the 342 health centers of the MoH in Lima; new cases diagnosed in hospitals are reported from their corresponding health center. We obtained incidence data from 2015 to 2017. TB diagnosis in the health centers is based on clinical suspicion (cough for more than 2 weeks, fever, night sweats) and/or microbiological confirmation. All reported new pulmonary TB cases (smear positive or negative) were used for this study. The populations of each district were provided by the National Institute of Statistics and Informatics (INEI) via the REtrieval of DATa for small Areas by Microcomputer (REDATAM) platform; incidence rates were computed at district-level per 100 000 habitants. Data handling and formatting were performed using R software.

#### Particulate matter 2.5 μm (PM_2.5_)

Air quality data from the Socioeconomic Data and Applications Center (SEDAC) by NASA was used [13]. SEDAC provides annual global surface concentrations (micrograms per cubic meter, μg/m^3^) of mineral dust and sea-salt filtered atmospheric PM_2.5_ [12, 14]. From 2015 to 2016, PM_2.5_ gridded datasets were provided at a spatial resolution of 0.01 degrees (~ 1.11 km at the equator) [14]. This product is the computation of aerosol optical depth (AOD) from multiple satellite instruments including the NASA Moderate Resolution Imaging Spectroradiometer (MODIS), Multi-angle Imaging Spectro Radiometer (MISR), and the Sea-Viewing Wide Field-of-View Sensor (SeaWiFS). SEDAC used a GEOS-Chem chemical transport model to relate the total column measure of aerosol to near-surface PM_2.5_ concentration and a geographically weighted regression (GWR) with global ground-based measurements to predict and adjust for the residual PM_2.5_ bias per grid cell in the initial satellite-derived values, previously validated [15]. We processed the raster data in Google Earth Engine (GEE) and summarized as the median PM_2.5_ value per year and district, scaled by 100.

#### Poverty level

Poverty level at the individual, household and neighborhood level was provided by INEI [16]. The downscaling of poverty indicators [17] were conducted using data from the National Census and the National Survey of Households (ENAHO), a specialized survey that includes detailed information about incomes and expenditures. Information on level of poverty was only provided for 2016, however, no significant relative changes were observed in previous estimates from 2013 [18]. Dalenius-Hodges method [19] was used to compute five poverty-stratum at individual, household and neighborhood levels. A dimension reduction was conducted using a principal component analysis (PCA) in order to assign a single poverty level value per district. Final poverty index was computed as the additive inverse of all principal components with an eigenvalue greater than 1, higher values reflecting an impoverished population and lower values a wealthy population.

### Spatial analysis

Spatial autocorrelation of TB cases, poverty, and PM_2.5_ were assessed using global Moran’s *I* statistics to describe the overall spatial dependence in the entire study area. In addition, local Getis-Ord Gi* statistic (a type of Local Indicator of Spatial Association - LISA) was used to identify local patterns and high-risk areas. A first-order queen contiguity-based weighted neighborhoods (districts with contiguous boundaries) were used for Moran’s *I* and Getis-Ord Gi*. Gi* statistic was categorized based on the sign (cold or hotspot) and percentile (90, 95, 99%) to prevent bias due to multiple and dependent tests [20]. Gi* statistic might be sensible to the fact that units near the edge would have fewer neighbors than those in the middle of the study area, known as edge-effect [21]. However, there is no consensus on the edge-effects on hotspot detection using areal data and their correction methods. In this study area, the median number of neighbors for districts located at the edges is four and five for those located in the middle. Under this small difference, the spatial analysis did not account for edge-effect correction, however, precaution would be taken if clusters were near the border of the study area.

Kendall’s W test was performed to evaluate the co-occurrence between cluster categories of TB cases, poverty, and PM_2.5_ as previously described [22]. Kendall’s W measure the concordance between two features, ranging from + 1 (complete agreement) to − 1 (no agreement) and was calculated for all combinations of two variables including TB cases (TB cases-poverty, TB cases-PM_2.5_) and the three at the same time. Legendre method [23] was used to compute a *P*-value based on Monte Carlo randomizations.

The spatial data processing, analysis, and visualization were performed using the ‘*spdep*’ and ‘*sf*’ packages; and Kendall’s W test were performed using the ‘synchrony’ package in R software.

### Statistical analysis

The Gini Index was calculated to assess the disproportioned distribution of cases in Lima districts. The Gini coefficient is a common measure of the inequality among values of a frequency distribution (TB cases). It is defined as a ratio with values between 0 (perfect equality) and 1 (perfect inequality). The Gini coefficient was computed using the ‘*ineq*’ package in R software.

A negative binomial generalized linear mixed model (GLMM) was created to assess the importance of poverty level and PM_2.5_ as drivers of spatial variation in TB incidence rate across Lima. A baseline model was formulated as follows:

$$ \log \left({\rho}_{st}\right)=\alpha +{\gamma}_{t(a)}+{\varphi}_s+{\upsilon}_s $$

Where the TB rate for each district and year (log(*ρ*_*st*_)) is modeled by 1) an intercept (α), 2) an exchangeable random effects for each year (γ_t(a)_) to account for interannual variation in TB over time (yearly random effect); and 3) spatially unstructured (*φ*_*s*_), and structured (*υ*_*s*_) random effects using a convolution prior that combines area-specific overdispersion and a neighborhood dependency structure [24]. Covariates (poverty level and PM_2.5_) were added to this model and model parameters were estimated within a Bayesian framework using Integrated Nested Laplace Approximation (INLA) [25], an alternative to Markov Chain Monte Carlo (MCMC) methods. Models were fitted using the ‘*INLA*’ package in R software.

## Results

### Baseline characteristics and TB incidence

A total of 28 381 new pulmonary TB cases were reported during the study period (2015–2017) with stable yearly rates. The incidence rate at district-level ranged between 18.8–214, 20.2–216, and 0–199 cases per 100 000 habitants in 2015, 2016, and 2017, respectively (Fig. 1b). The average PM_2.5_ concentration at district-level was slightly higher in 2016 (29.5 μg/m^3^) than in 2015 (26.2 μg/m^3^). The average PM_2.5_ was highly heterogeneous across districts in Lima, ranging between 13.8–40.4 μg/m^3^ in 2015, and 16.5–44.8 μg/m^3^ in 2016.

### Spatial clustering and co-occurrence

**Fig. 2:**
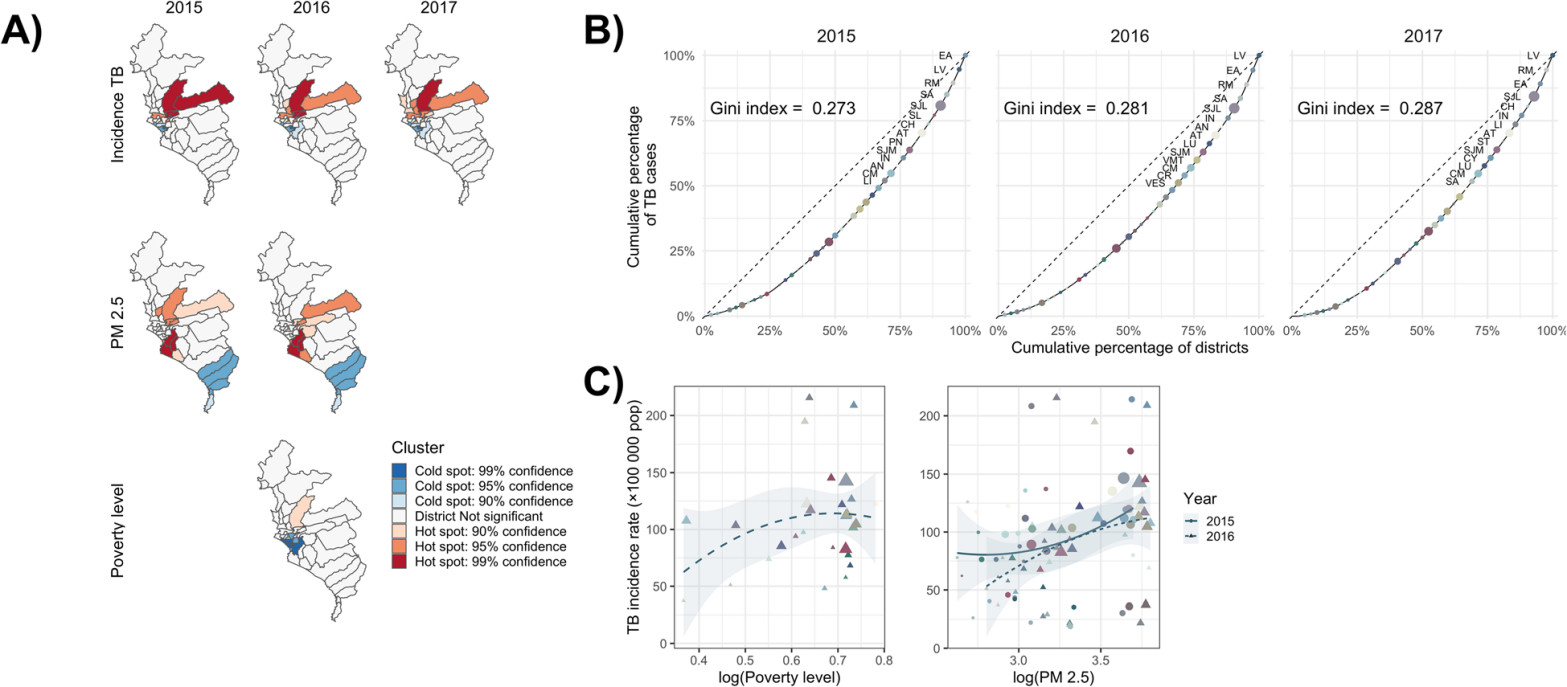
Clustering, GINI index and distribution relative to covariates of tuberculosis in Lima 2015–2017. **a** Local *Getis-Ord G*_i_^*^ clusters of TB incidence, poverty, and PM_2.5_ in Lima 2015–2017. **b** Lorenz curve of TB cases in Lima 2015–2017. **c** Incidence of TB relative to PM_2.5_ concentration and poverty in Lima 2015–2017. x-axis in logarithmic scale. Each color represents a district. Point size scaled to population size

An overall strong spatial autocorrelation was observed during the 2015–2017 period for TB cases (Moran’s *I* range: 0.24–0.36, *P* < 0.01), PM_2.5_ (Moran’s *I* range: 0.55–0.56, *P* < 0.001), and poverty level (Moran’s *I* = 0.353, *P* = 0.003) (Supplementary Fig. 1). Stable high-risk clusters of TB cases were observed in the central-east part of Lima and low-risk clusters in the south-west (Fig. 2a). High-risk clusters of PM_2.5_ were detected in the south and central-east of Lima, with low-risk clusters located in the south. Furthermore, cold spots of poverty level (cluster of wealthy districts) were located in the central-west. Statistically significant co-occurrence of clusters (seven categories - cold spot 99% confidence, cold spot 95% confidence, cold spot 90% confidence, not significant, hot spot 99% confidence, hot spot 95% confidence, hot spot 90% confidence) of TB cases and PM_2.5_ (Kendall’s W = 0.596; *P* = 0.046), TB cases and poverty level (Kendall’s W = 0.4714; *P* = 0.003), and the three variables combined (Kendall’s W = 0.4606; *P* = 0.001) were observed.

### Mixed-effects models and inequity indexes

**Fig. 3:**
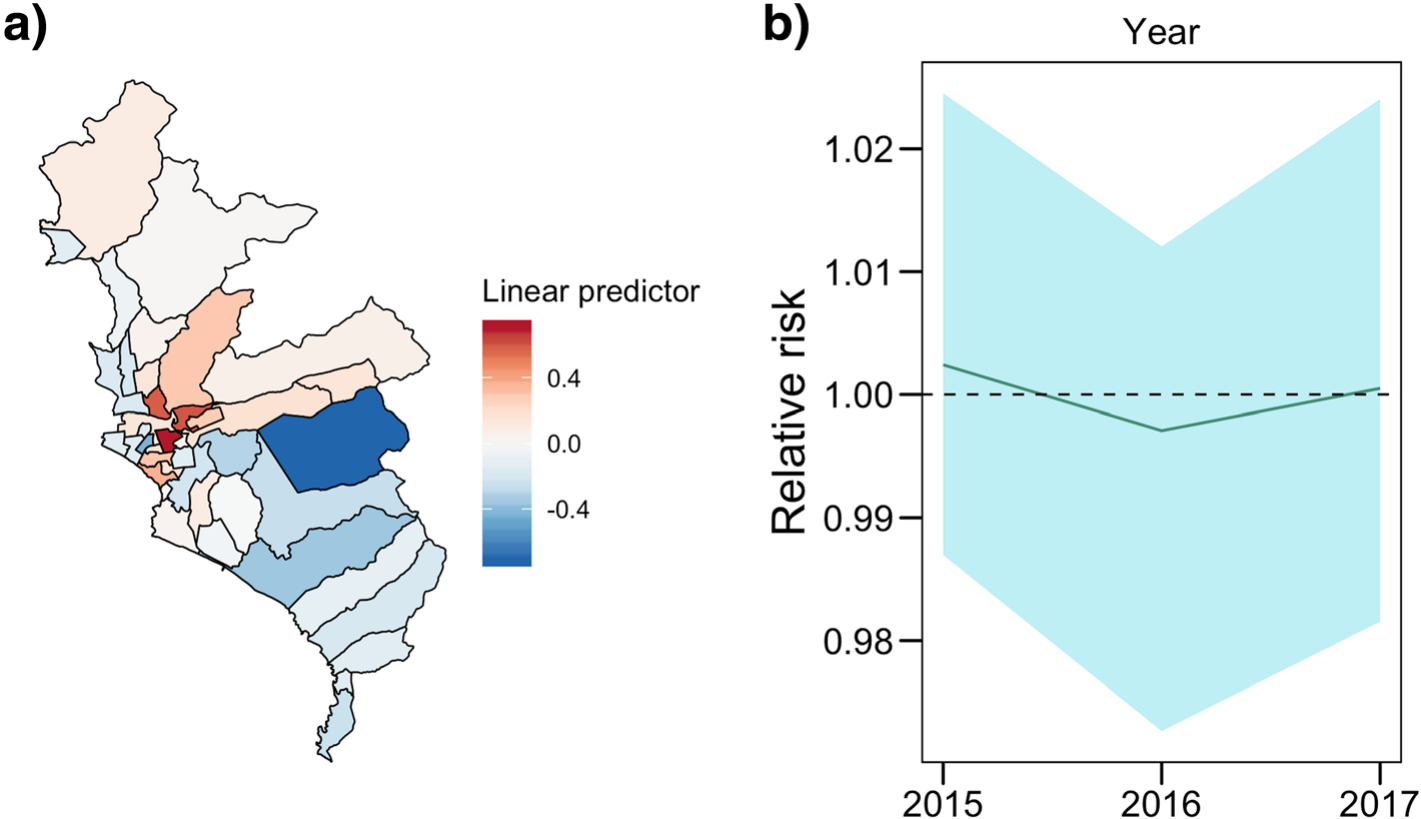
Contribution of spatial and temporal random effects to tuberculosis rate. **a** Marginal posterior mean of the combined spatially structured and unstructured random effects at the linear predictor scale of seasonal-spatial models. **b** Non-linear shape of year random effect

A moderate concentration of TB cases among Lima districts were observed in the study period (Gini Index range = 0.27–0.29) (Fig. 2b). The general trends of TB cases with PM_2.5_ and poverty level are present in Fig. 2c. The slight decrease in TB incidence, in spite of the increasing poverty index, can be explained by the efforts of the MoH and National TB Program towards TB control among low-resource districts. The spatio-temporal Bayesian mixed-effects negative binomial multivariate regression shows that PM_2.5_ (adjusted relative risk [ARR] = 1.31; 95% credible interval [*CI*]: 1.17–1.50) and poverty level (ARR = 1.14; 95% *CI*: 1.11–1.17) were associated with TB incidence rate in Lima, accounting for the spatio-temporal structure of the districts (Table 1); spatial and temporal random effects are shown in Fig. 3.

**Table 1:** Estimates of Spatio-temporal Bayesian mixed-effects negative binomial regression

Variables	Unadjusted			Adjusted		
	RR	Std. Dev.	95% credible interval	RR	Std. Dev.	95% credible interval
PM_2.5_	1.306	0.063	1.154–1.484	1.318	0.064	1.166–1.504
Poverty level	1.142	0.013	1.112–1.173	1.142	0.013	1.113–1.172

*RR* Relative Risk, *PM*_*2.5*_ fine particulate matter

## Discussion

This study describes the skewed spatial distribution of TB cases in Lima. A strong spatio-temporal clustering of poverty, environmental fine particulate matter PM_2.5_ and incident TB cases was observed during the study period. Importantly, clusters of elevated PM_2.5_ concentrations, poverty, and high TB incidence significantly co-occur during the 2015–2016 period, suggesting that socio-economic determinants and environmental conditions interplay as important components of transmission and clustering of TB in this setting.

Lima is the capital with one of highest ambient air pollution levels in Latin America [26]; however, its burden on health remains largely unknown. Previous studies in Peru have explored the effect of air pollution as a determinant of asthma [27]. The most important effects of air pollution manifest in the upper respiratory tract where it alters immune response [8], contributing as a susceptibility factor for various respiratory diseases [28]. One study showed impaired the expression of CD69, IFN-γ and TNF-α when human peripheral blood mononuclear cells are exposed to *Mycobacterium tuberculosis* [29]. Likewise, exposure to air pollution has been linked to many substantial adverse effects on human health; in particular people with chronic respiratory diseases such as asthma and chronic obstructive pulmonary disease.

These findings are consistent with previous studies supporting the hypothesis of air pollution as an environmental determinant of TB [30]. One study in Jiangsu (China) showed association between TB and long-term pollution exposure (using PM_2.5_, PM_10_, SO_2_ and NO_2_ measurements); similar findings were found in North Carolina for long-term pollution exposure (PM_10_ and PM_2.5_) [7]. Short-term exposure to outdoor pollution also seems to be a factor for active TB [31] and can also increase the risk for TB infection [32]. Poverty condition also seems to be a key variable for unhealthy concentrations of PM_2.5_ and high-risk of TB alike. Although poverty has been proven to be a driver of the TB epidemic, it is also an important determinant of outdoor and indoor air pollution. The concentrations of the pollutants stated above are often highest largely in the urban areas of low- and middle-income countries [33]. Rapid rural-urban migration, as experienced in Lima, has created overcrowded districts characterized by poverty and increased air pollution concentrations [34]. The inequalities of TB are seldom looked at from an environmental perspective; however, these findings suggest the complex interaction between socio-economic factors and environment pollution in the transmission of TB in Lima.

Some limitations are acknowledged in this study. First, the high spatial resolution of the NASA-SEDAC PM_2.5_ estimates could not be fully harnessed due to the coarse spatial resolution of the TB data at the district level. Some districts present a high heterogeneity in PM_2.5_ between sub areas that were not included in this study. Additionally, NASA-SEDAC estimates lack a high temporal resolution; only yearly estimates were provided and seasonal variations in PM_2.5_ have been reported [35]. Also, the effect of long-term exposure to PM_2.5_ and other markers such as PM_10_, SO_2_ and NO_2_ were not evaluated due missing information. Finally, TB reporting system (SIGTB) was established in 2015 and NASA-SEDAC only provided data until 2016, resulting in a short study period to observe the relation between PM_2.5_ and TB incidence.

## Conclusions

This study describes the co-occurrence of clusters of elevated concentrations of air pollution (measured by PM_2.5_), poverty and high-risk areas of TB in Lima. These findings support previous studies and address the interplay of socio-economic and environmental drivers of TB incidence that will help to tailor TB control interventions. Further studies should be done to confirm these results at the individual level.

## Supplementary information

**Additional file 1 : Figure S1.** Global Moran’s *I* of tuberculosis incidence, poverty, and PM_2.5_ in Lima 2015–017.

## Data Availability

All datasets are presented in the main paper and the supplementary materials. Data sharing is not applicable to this article as no datasets were generated or analyzed during the current study.

## Abbreviations

TB: Tuberculosis
PM_2.5_: Fine particulate matter
MoH: Ministry of Health
NASA: National Aeronautics and Space Administration
INEI: National Institute of Statistics and Informatics
REDATAM: Data Recuperation for Small Areas by Microcomputers
SEDAC: Socioeconomic Data and Applications Center
AOD: Aerosol Optical Depth
MODIS: Moderate Resolution Imaging Spectroradiometer
MISR: Multi-angle Imaging SpectroRadiometer
SeaWiFS: Sea-Viewing Wide Field-of-View Sensor
GWR: Geographically Weighted Regression
GEE: Google Earth Engine
ENAHO: National Survey of Households
PCA: Principal Component Analysis
LISA: Local Indicator of Spatial Association
GLMM: Generalized linear mixed model
INLA: Integrated Nested Laplace Approximation
MCMC: Markov Chain Monte Carlo
SO_2_: Sulfur dioxide
NO_2_: Nitrogen dioxide
