# Supplementary figures and images for: Spatio-temporal co-occurrence of hotspots of tuberculosis, poverty and air pollution in Lima, Peru
Source: Infect Dis Poverty. 2020 Mar 24;9:32. doi: 10.1186/s40249-020-00647-w (PMC7092495; doi:10.1186/s40249-020-00647-w)

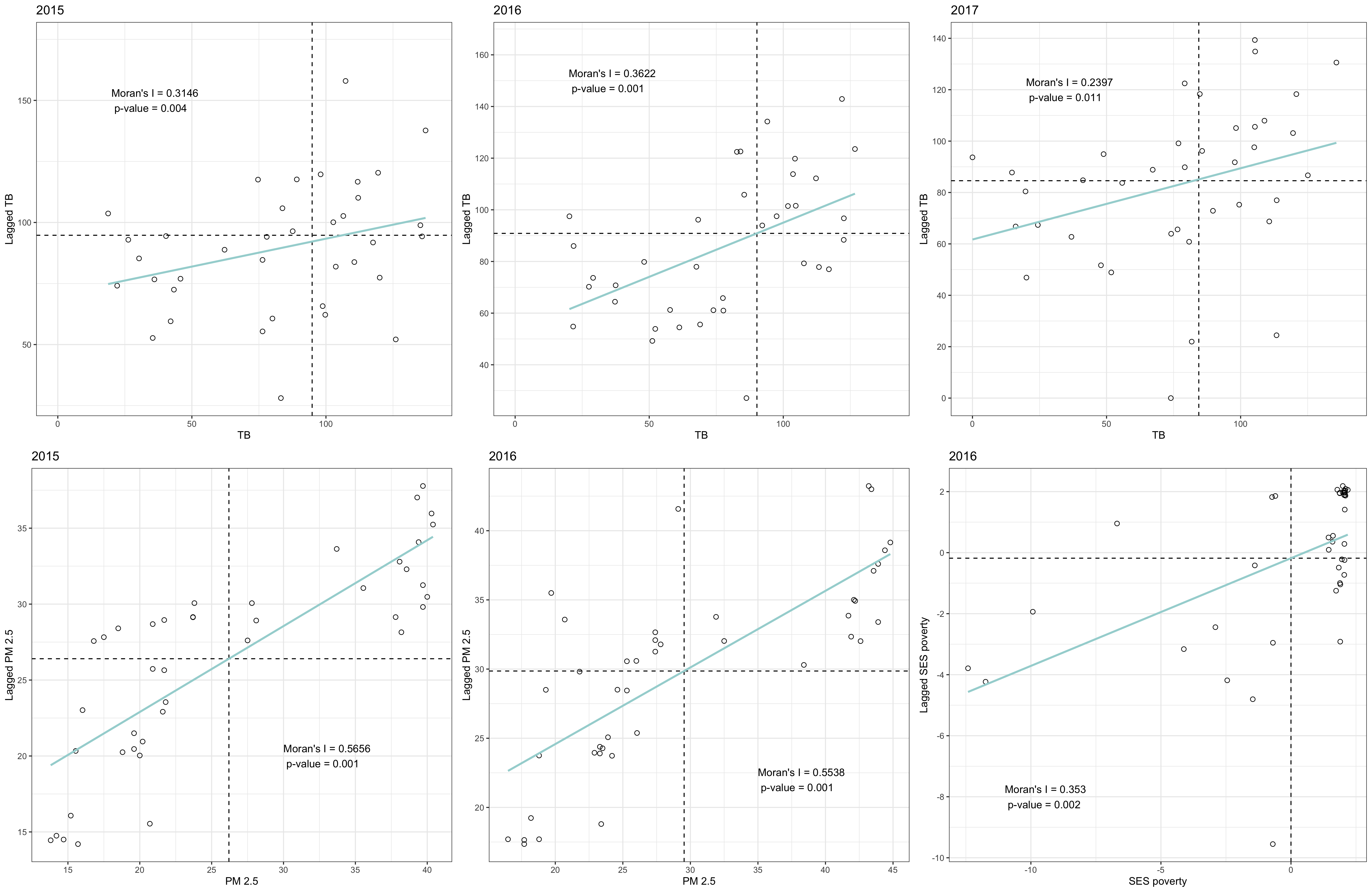

Supplement: Supplementary file 1 — Additional file 1 : Figure S1. Global Moran’s I of tuberculosis incidence, poverty, and PM2.5 in Lima 2015–017. [file 40249_2020_647_MOESM1_ESM.png]
